# Management of Hypertriglyceridemia Induced Acute Pancreatitis

**DOI:** 10.1155/2018/4721357

**Published:** 2018-07-26

**Authors:** Rajat Garg, Tarun Rustagi

**Affiliations:** ^1^Department of Internal Medicine, Cleveland Clinic, Cleveland, OH, USA; ^2^Division of Gastroenterology and Hepatology, University of New Mexico, Albuquerque, NM, USA

## Abstract

Hypertriglyceridemia is an uncommon but a well-established etiology of acute pancreatitis leading to significant morbidity and mortality. The risk and severity of acute pancreatitis increase with increasing levels of serum triglycerides. It is crucial to identify hypertriglyceridemia as the cause of pancreatitis and initiate appropriate treatment plan. Initial supportive treatment is similar to management of other causes of acute pancreatitis with additional specific therapies tailored to lower serum triglycerides levels. This includes plasmapheresis, insulin, heparin infusion, and hemofiltration. After the acute episode, diet and lifestyle modifications along with hypolipidemic drugs should be initiated to prevent further episodes. Currently, there is paucity of studies directly comparing different modalities. This article provides a comprehensive review of management of hypertriglyceridemia induced acute pancreatitis. We conclude by summarizing our treatment approach to manage hypertriglyceridemia induced acute pancreatitis.

## 1. Introduction

Gallstones and alcohol abuse are the two most common causes of acute pancreatitis (AP). Hypertriglyceridemia is an uncommon but a well-established etiology of acute pancreatitis, with a reported incidence of 2-4% [1–3]. National cholesterol Education Program ATP III categorizes triglyceride (TGs) level as normal (<150), borderline high (150-199), high (200-499), and very high (>500 mg/dL) (1 mmol = 88.5736 mg/dL) [4]. Typically, TG levels >1000 mg/dL have been associated with AP; however, the level above which AP may occur is unknown and varies with individuals. There are various epidemiological studies that tried to determine the appropriate cut-off for TG level to cause AP. In a study of 129 patients with severe HTG, 26/129 (20.2%) patients experienced at least one episode of AP [5]. Data from European population studies also show AP incidence of 10-19% of patients with severe HTG (>1000 mg/dL) [3]. Sandhu et al. reported that HTG is unlikely to be cause of acute pancreatitis at TGs levels <1771 mg/dL as none of the 95 patients had AP when TGs were less than 1771 [6]. In a large population study, adjusted hazard ratio for AP was 3.20 (CI, 1.99-5.16) in group with TGs levels > 500 when compared with group with TGs levels < 150. Moreover, there was 4% increase of incidental AP for every 100 mg/dL increase in TG concentration (after adjustment for covariates and other variables) [7]. These studies are in alignment with current recommendation that TG < 1000 mg/dL is unlikely to cause acute pancreatitis [2, 3, 8].

## 2. Etiology

The etiology of HTG can be broadly divided into 2 categories: primary and secondary. While primary one causes more severe HTG, it is the interplay of both primary and secondary factors that leads to severe HTG. Severe HTG is commonly seen with familial chylomicronemia syndrome (FCS), primary hypertriglyceridemia, and mixed hypertriglyceridemia also known as Fredrickson Type I, IV, and V, respectively, in pregenomic era. FCS (I) and mixed hypertriglyceridemia (V) lead to more severe HTG and often present early, whereas primary hypertriglyceridemia (IV) presents in adulthood usually precipitated by secondary factor. Common genetic defects leading to severe HTG include lipoprotein lipase deficiency, LPL gene mutation, and Apolipoprotein C II deficiency in addition to mutations in GPIHBP1 (glycosylphosphatidylinositol-anchored high-density lipoprotein binding protein 1), LMF1, and other genes involved in lipoprotein generation and metabolism [9]. A severe HTG is likely to have monogenic pattern where mild to moderate HTG is likely to have polygenic inheritance [9].

One or more secondary factors are commonly seen with patients with HTG-AP as reported by multiple studies [5, 6, 10–12]. Secondary factors associated with HTG include obesity, alcohol abuse, uncontrolled diabetes mellitus, hypothyroidism, chronic renal failure, and drugs like estrogen, corticosteroids, and retinoids. A detailed history to elicit the underlying factors should be performed in patients with HTG-AP and genetic screening should be considered if indicated, based on their family history. More recently, immunological disorders including autoimmune hypertriglyceridemia caused by antibodies to LPL, Apo-CII, or GPIHBP1 have been reported in patients without genetic mutation or secondary factors [13–15].

## 3. Pathophysiology

The exact mechanism of HTG causing AP is not clearly understood. Most accepted theories are based on animal models which describe metabolism of excessive TGs by pancreatic lipase to free fatty acids (FFA) leading to pancreatic cell injury and ischemia [2, 8]. Hyperviscosity from excessive TGs in pancreatic capillaries leading to ischemia has also been proposed, but why this ischemia only occurs in pancreas and not in other organs is unknown. Specific genetic mutations such as CFTR and ApoE gene mutation have been associated with HTG-AP [3, 8]. It is likely that HTG induced AP result from complex interplay between multiple factors with varying contribution in individual patient. Further research is needed to elucidate the exact pathogenesis of HTG-AP.

## 4. Clinical Presentation

HTG-AP is diagnosed as any other acute pancreatitis with presence of 2 of 3 factors: characteristic abdominal pain, elevation of pancreatic enzymes over 3 times the upper limit of normal (ULN), and radiologic evidence of acute pancreatitis. Amylase level at presentation could be normal and should be interpreted with caution due to calorimetric interference of lipaemic serum; and repeat amylase levels should be performed with severe dilutions [8, 16, 17]. Serum TGs level should be measured as soon as the symptoms start as serum TG levels can rapidly trend down with fasting. There are conflicting data on severity of HTG-AP compared to AP from other causes with few studies reporting a more severe course with complications while others report no difference in the severity of acute pancreatitis [18–21]. In a recent study including 121 patients evaluating natural course of HTG-AP, local complications were higher in patients with TG ≥ 1000mg/dL, and chronic pancreatitis was reported in 17.8% of patients [12]. Moreover, when compared with AP from other causes the needs for ICU admission (39% versus 16%, P ≤ 0.001), SIRS (56% versus 28%, P ≤ 0.03), and persistent organ failure (23% versus11%, P ≤ 0.05) were significantly higher in HTG-AP cohort. However, a study of 43 patients reported no difference in severity, based on APACHE II scores or complications in patients with and without HTG, although the study has been criticized for including subjects with low mean TG levels [11].

Elevated serum TGs levels have also been associated with persistent organ failure in patients with AP irrespective of underlying etiology [22]. Moderate HTG (odds ratio (OR), 2.6; P ≤ 0.04) and severe/very severe HTG (OR, 4.9; P ≤ 0.009) were independently associated with persistent organ failure, on multivariate analysis after controlling for age, gender, body mass index, diabetes, and alcohol etiology [22]. In a retrospective Spanish study of 19 patients comparing HTG-AP and gallstone-induced AP, there was a trend towards longer hospital stay (24 versus 7.6 days) and more complications in patient with HTG-AP but the data did not reach statistical significance [23].

In a retrospective study of 224 patients with HTG-AP, incidence of severe AP in patients with TGs > 1000 mg/dL was higher as compared to patients with TGs 500-1000 mg/dL (*P* ≤ 0.045) [24]. However, there was no difference in local and systemic complications between the 2 groups. There was a statistical significant difference in severity of HTG-AP as compared to biliary AP (*P* ≤ 0.001) and alcoholic AP (*P* ≤ 0.007). Systemic complications with HTG-AP were also significantly higher than biliary AP (*P* ≤ 0.001).

Deng et al. reported severe HTG-AP (TGs > 500 mg/dL) was associated with higher 24 hr APACHE II score, increased incidence of renal failure, shock, infection, and overall mortality (31.1 versus 9.1, P < 0.01) when compared with severe AP from other causes [25].

In another retrospective study of 144 patients, the authors reported statistically significant higher incidence of local complications mainly acute pancreatic fluid collections (APFC) on CT scan (69% versus 45%, p = 0.002), moderate to severe pancreatitis (74.3% versus 50%, p ≤ 0.005), and more than 3 organ failures ( 10% versus 0%, p = 0.008) in patients with high TGs levels >2648 mg/dL as compared to the group with low TGs levels (1000-2648 mg/dL) [26]. Severity was defined according to revised Atlanta classification. There was trend towards increased mortality (7 versus 1, p = 0.07), ICU stay, and length of hospitalization but the differences were not statistically significant. The cut-off for TGs level was arbitrary. This study also demonstrated a trend towards severe AP in patients with higher TGs levels.

To date, the largest study comparing HTG-AP (17.5%) versus non HTG-AP (82.5%) including 3558 patients reported statistically significant (all P < 0.01) higher incidence of pancreatic necrosis (28.3% versus 18.1%), infected pancreatic necrosis (6.1% versus 3.7%), organ failure (35.8% versus 29.1%), and persistent organ failure (24.4% versus 16.5%) [27]. There was positive correlation between TGs level <24 h after onset and disease severity (r = 0.26, P < 0.01) with serum TG levels <24 h of onset 9.38 ± 9.00 mmol/L, 11.90 ± 9.02 mmol/L, and 16.47 ± 11.75 mmol/L in patients with mild, moderate, and severe HTGP, respectively.

Recent studies demonstrate trend towards severe pancreatitis in patients with HTG-AP when compared with non-HTG pancreatitis. Among those with HTG-AP, the patients with higher TGs levels appear to have more severe hospital course with a higher incidence of complications (35-69%) and organ failure (20-35%). There is also a trend towards prolonged hospitalization and higher mortality.

## 5. Management

As in any patient with acute pancreatitis, conservative treatment including aggressive intravenous hydration, initial bowel rest, and pain control should be initiated as soon as diagnosis is suspected. Patients should be risk-stratified based on the severity of acute pancreatitis to guide appropriate management. Patients with Balthazar grade E and/or APACHE II score ≥ 8 should be considered as severe pancreatitis and potentially managed in intensive care unit [28–30]. In addition, several treatment modalities have been described specifically for management of hypertriglyceridemic pancreatitis.

## 6. Insulin and Heparin

Insulin therapy has been used for more than a decade to lower TG level along with heparin. While there are many case reports and series demonstrating TG lowering effect, there are no comparison studies evaluating insulin versus conservative therapy [31–33]. Insulin activates lipoprotein lipase (LPL) activity which in turn accelerates chylomicron degradation thus lowering TGs levels [34]. Insulin will also rest pancreatic tissue and may improve immunoparalysis via upregulating the expression of human leukocyte antigen on monocytes and decreasing cell apoptosis [35]. Insulin lowers TGs levels by 50-75% over 2-3 days [34]. Cases of successful management of HTG-AP have been reported even in nondiabetic patients without concomitant serum glucose elevation (75% drop in serum TGs in 24 hours) [31, 36–38]. In a recent meta-analysis evaluating the effects of insulin therapy on outcomes of severe acute pancreatitis, intensive insulin therapy was found to lower APACHE II score after 72 hours of treatment (weighted mean difference (WMD) = -3.80, 95% CI [-4.88,2.72], p < 0.00001) and shorten the length of hospitalization (WMD = -12.13, 95% CI [-15.48,8.78], p < 0.00001); however, only 3 studies with small sample size were included in this meta-analysis [35].

Heparin releases stored lipoprotein lipase from the endothelial cell thus lowering TGs levels. Combination of insulin and heparin has been used to lower TGs level in case reports and case series with mean decrease of TGs level by 50% within 24 hours [17, 31, 39]. There is a concern of rebound hypertriglyceridemia as long term or continuous heparin infusion has been shown to deplete LPL, leading to reduction of chylomicrons catabolism and increase in TGs levels. A case of pregnant women who developed HTG-AP after long term use of heparin has also been reported [40]. In a study of 10 healthy subjects on heparin infusion, LPLs levels initially peaked, eventually attaining plateau levels after 6 hours of heparin infusion which was 15% of the peak levels [41]. This also demonstrates role of heparin in recruitment of stored LPL until there is balance between LPL synthesis and hepatic uptake. Low molecular weight heparin has also been shown to lower LPLs level similar to conventional unfractionated heparin infusion [42].

In summary, both insulin and heparin have been reported to be safe and effective acute TGs lowering therapy but, due to concern of rebound hypertriglyceridemia and risk of hemorrhage into the pancreas during acute attack on continuous heparin infusion, heparin should preferably be avoided. Although there are no randomized trials comparing insulin and conservative therapy, insulin at least appears to be safe and effective therapy for HTG-AP even in nondiabetic patients from the multiple reports. Frequent blood glucose checks should be done to avoid undetected hypoglycemia and if needed, dextrose infusion should be started to maintain euglycemia until TGs levels are < 1000.

## 7. Plasmapheresis (PEX)

Plasmapheresis (PEX) rapidly removes TGs and chylomicron from the circulation removing the inciting factor and halting the further inflammation and damage to the pancreas. PEX lowers the lipid levels drastically within hours compared to conservative therapy that usually takes several days to achieve the same reduction in TG levels. Most patients require only one session of PEX as it is reported to lower TG levels by 50-80%. It has been postulated that PEX improves HTG-AP outcomes not only by lowering TG levels but also by removing proinflammatory markers and cytokines to downregulate the inflammatory process in HTG-AP [43]. Although multiple case series report the benefit of PEX in management of HTG-AP, the only prospective study to date with a historic control (60 versus 34 patients) failed to show any mortality benefit compared to conservative management. Delay in initiating PEX was thought to be a potential reason for this lack of difference in mortality [44]. However, another recent large retrospective study including 111 patients treated with PEX also found no mortality benefit in patients who received early PEX (within 36 hours) versus late PEX ( >36 hours) for HTG-AP [19]. The authors also reported a significantly lower mortality in patients who received citrate anticoagulation (1%) during PEX compared to heparin anticoagulation (11%) (P = 0.04); however, 24% patients in heparin group had severe pancreatitis as compared to citrate group (14%) but the difference was not statistically significant (P = 0.19).

There is limited data on use of PEX to prevent further episodes of pancreatitis in patients with recurrent HTG pancreatitis [45]. Some authors recommend albumin as replacement fluid of choice for PEX to avoid complications associated with plasma infusion [19].

A multicenter retrospective study reported possible role of PEX in treatment of patients failing conservative treatment including diet and lipid lowering agents [46]. They reported mean change of -67% in TGs level in 17 patients with HTG, and 12 out of 17 also had history of pancreatitis. Authors concluded that since presence of HTG is itself a risk factor for recurrent pancreatitis and cardiovascular disease, PEX should be considered even in outpatient settings in patients with symptomatic and resistant hypertriglyceridemia. PEX has also been reported to prevent further episodes in patients with recurrent pancreatitis with 67% reduction reported in case series of 6 patients [47].

While PEX appears to be most effective in rapid lowering TGs level, treatment is not without adverse effects. Plasma infusion itself is associated with risk of infections and allergic reactions to donor plasma. Moreover, PEX requires central venous access which itself predisposes to another set of complications including but not limited to infections, hemorrhage, injury to blood vessel, and thrombosis. While the risk is real, reported incidence of adverse effects is low (5.7%) mainly blood access problems, tingling, hypotension, and urticaria [48]. Careful risk assessment should be performed before initiating therapy. In addition, PEX is costly and not widely/easily available.

In conclusion, Due to the paucity of randomized trials or high-quality comparator, no specific recommendations can be made regarding the starting points or end points but it seems reasonable to initiate the PEX in patients with HTG-AP as early as possible (preferably within 36 hours) to halt further inflammation and necrosis of pancreatic tissue. Most studies have utilized TGs level <500 to be end point of therapy. PEX should be considered in patients with severe HTG-AP with APACHE II score ≥8 since mortality rates are high and reported to be around 10-39% [49].  Table 1 summarizes those papers reporting outcomes employing PEX for treatment of HTG-AP in more than 5 patients. American Society for Apheresis considers HTG-AP as Grade 2C recommendation (individualized decision is necessary, unclear role of therapy) [50]. Albumin should be utilized as choice of replacement fluid and citrate should be preferred over heparin. In patients receiving citrate anticoagulation, ionized calcium levels should be monitored and replaced periodically. There is no consensus on end points of treatment as there might be some benefit in targeting symptoms resolution as compared to specific TGs levels [51].

## 8. Combined Blood Purification Therapy (CBPT)

CBPT is a two-step approach for management of acute severe pancreatitis involving plasmapheresis and continuous venovenous hemofiltration. Coupled plasma filtration adsorption combined with CVVH has been shown to improve mortality and lowering of inflammatory markers in severe acute pancreatitis irrespective of etiology of pancreatitis [52]. Sequential blood purification therapy involving plasma exchange and CVVH has been shown to improve 28-day mortality, ICU stay, and time to target lipid level in patients with severe HTG-AP [53]. Study patients with severe HTG-AP (Marshall Criteria > 2, Serum TGs > 1000) were treated with plasma exchange initially with goal TGs < 500 followed by CVVH until resolution of symptoms and Marshalls score < 2 and were compared with control group treated with only CVVH. There were no statistical differences in rates of systemic or local complications. Despite the limitations of small sample size, observational design, and possible bias, this study highlights the potential role of CBPT in severe acute pancreatitis with possible mortality benefit. More studies with better design are required before any strong recommendations can be made for CBPT.

## 9. High-Volume Hemofiltration (HVHF) and Hemoperfusion (HP)

Continuous venovenous filtration is commonly used for severe acute pancreatitis and has shown good results including mortality benefit; however roles of hemofiltration in HTG-AP have been recently explored. Mao et al. in 2003 studied the role of hemofiltration in HTG-AP by employing hemofiltration with hemoadsorption in patient with HTG induced severe acute pancreatitis (SAP). Adsorption of triglyceride and hemofiltration blood purification was performed using Diapact CRRT machine (B. Braun Co., Germany) and polysulfone filters (cut-off point 30 000 Dalton, AV 600S, Fresenius Medical Care). There was statistically significant reduction of serum TGs, IL-10, and APACHE II score with no mortality in severe acute pancreatitis patients and 33% mortality in fulminant acute pancreatitis [51]. This was the first study to evaluate the potential role of hemofiltration and hemoadsorption in HTG-AP. In 2011, Tang et al. reported a case of successfully treated acute severe HTG-AP with hemoperfusion and CVVHD [54]. High-volume hemofiltration has been shown to improve clinical outcomes in severe acute pancreatitis even before appearance of kidney injury [55]. Furthermore, a recent study reported a statistically significant improvement in APACHE II score, SOFA score, HR, TGs level, and cytokines level including IL-1, IL-2, IL-6, IL-8, IL-10, and TNF-a in patients with severe HTG-AP treated with hemofiltration and hemoperfusion as compared to the control group [56]. Patients underwent 2 cycles of HVHF and HP of 24 hours each with 2 hours of HP within 48 hours of presentation. An AN69 hemofilter (1.6 m2 surface area, 35-KD limit; Baxter Healthcare Corp., Deerfield, IL, USA, http://www.baxter.com/) was used and HP was carried using a synthetic resin cartridge (HA-330; Zhuhai Lizhu Group, Biological Material Co., Ltd., China) installed before the hemofilter. This study reported improved clinical outcomes but was limited by small sample size, single center, and no reporting of mortality difference. This was followed by a randomized controlled trial to evaluate the early HVHF versus insulin and heparin in patients with severe HTG-AP [57]. Continuous venovenous hemofiltration (PrismaFlex; Gambro, Lund, Sweden) was conducted as soon as possible after admission using a synthetic AN69HF membrane created with acrylonitrile and sodium methallyl sulfonate copolymer (PrismaFlexM100; Gambro) with goal TG level <5.65 mmol/L (500 mg/dL). Significant improvements of APACHE score (p ≤ 0.028) and TGs levels (p ≤ 0.01) were reported on Day 1 but no significant differences were present on Day 2 (p = 0.09 & 0.103) and Day 5 (p = 0.25 & 0.417). In addition, HVHF was associated with persistent organ failure [50% versus 20.6%; risk ratio (RR), 2.42; 95% CI, 1.15-5.11; P≤0.01] mainly respiratory failure and was associated with double the cost of hospitalization compared to conservative management group. There was no difference in levels of CRP, procalcitonin, and other complications of acute pancreatitis. HVHF was more effective in lowering TG level faster (approximately 9 hours) compared to insulin and heparin groups (48 hours). No difference of clinical outcomes was observed in HVHF group. All three studies are summarized in Table 2.

There are certain reasons for such variable results in the studies. First there was no uniform definition of severe acute pancreatitis in patients enrolled in the studies, some used APACHE score of >15, and others used SIRS >2 as inclusion criteria in the studies. Second, the study that showed benefit performed HVHF with HP for 48 hours regardless of TG levels but another study had TG level directed therapy that was achieved in 12 hours. Third, HP also played a role in removal of TG and cytokines leading to more favorable outcomes in HVHF-HP group. HVHF has role in severe AP with possible inclusion criteria of APACHE score >15, provided HF can be started within 72 hours of initial presentation and continued for at least 48 hours. Full benefit of HVHF is derived not only from TG lowering therapy but also from removal of proinflammatory cytokines. In conclusion, HVHF should be considered in patients with severe HTG-AP with APACHE score > 15 where plasmapheresis is not available. More studies evaluating the role of HVHF and HP are required as a possible alternative to PEX.

## 10. Pharmacologic Therapy

Once patient can tolerate oral intake, adjunctive lipid lowering therapy should be started with the goal of achieving long term TGs control. Consultation with dietician should be sought and patient should be counseled on the importance of low fat diet to prevent recurrence. Lifestyle changes including better diabetes control and alcohol abstinence should be reinforced. Lipid electrophoresis should be done to ascertain the type of hypertriglyceridemia for any genetic component after secondary causes are ruled out.

According to ATP 3 guidelines, fibrates remain the drug of choice for severe HTG (TGs > 500 mg/dL) with niacin as adjunctive therapy [4]. Fibrates, statins, niacin, and omega three fatty acids have been shown to reduce TGs levels by 36.3%, 10% to 18%, 20%, and 25 to 33.8%, respectively [65–67]. Fibrates are the most effective in lowering TGs levels in all antihyperlipidemia drugs. Common adverse effects associated with fibrates are myopathy, cholelithiasis, and reversible increase in creatinine. Fibrates if combined with statin increase risk of rhabdomyolysis two times as compared to fibrate monotherapy but overall risk is very low (2.8/10000 person-years) [68]. Gemfibrozil is associated with higher risk of myopathy as compared with fenofibrate and should not be combined with statin [69]. Interestingly, fibrate therapy was shown to be associated with increased risk of pancreatitis compared with placebo (RR = 1.39, CI, 1.00-1.95; p = 0.053) in a meta-analysis but those studies only included patients with relatively lower (200-499 mg/dL) TGs levels which are not associated with pancreatitis [70]. High dose niacin therapy is also effective in lowering TGs level but its use is limited by frequent adverse effects (flushing (30% of patients), pruritus, gastrointestinal disorders, hyperglycemia, blurring of vision, myopathy, and elevation of liver enzymes) seen at high doses. Moreover, it has been associated with increase in plasma glucose levels and worsening diabetes [71]. Omega 3 fatty acids are effective drugs with good safety profile. In a meta-analysis, they were well tolerated without any serious adverse effects and risk of discontinuation is comparable to placebo group [72].

Combination of medium chain triglycerides and omega 3 fatty acids has been reported to lower TGs levels by 67% over one-week period [73]. Medium chain triglycerides do not form chylomicrons when absorbed and prevent postprandial hyperchylomicronemia. Diet containing total fat < 15% should be started to prevent hyperchylomicronemia.

Novel pharmacologic treatments for severe HTG have been described as mainly microsomal transfer protein (MTP) inhibitor Lomitapide and gene therapy for hereditary hypercholesterolemia and LPL deficient individuals [74]. Newer triglyceride-lowering modalities under evaluation and at different stages of development include gene therapy for lipoprotein lipase deficiency (alipogene tiparvovec, and antisense oligonucleotides against mRNA for apolipoproteins B (mipomersen) and C3 (volanesorsen, ISIS 304801), diacylglycerol acyltransferase-1 (pradigastat), and a monoclonal antibody against angiopoietin-like protein 3 (REGN1500) [75]. There are existing data on biochemical efficacy but long term safety data is lacking.

In summary, fibrates remain the drug of choice for long term TGs control with omega 3 fatty acid as reasonable second choice. Niacin and statins can be added but, to combined adverse effects, they are not well tolerated. More treatment options of hereditary lipoprotein syndromes are currently being explored.

## 11. Conclusion

**Figure 1:**
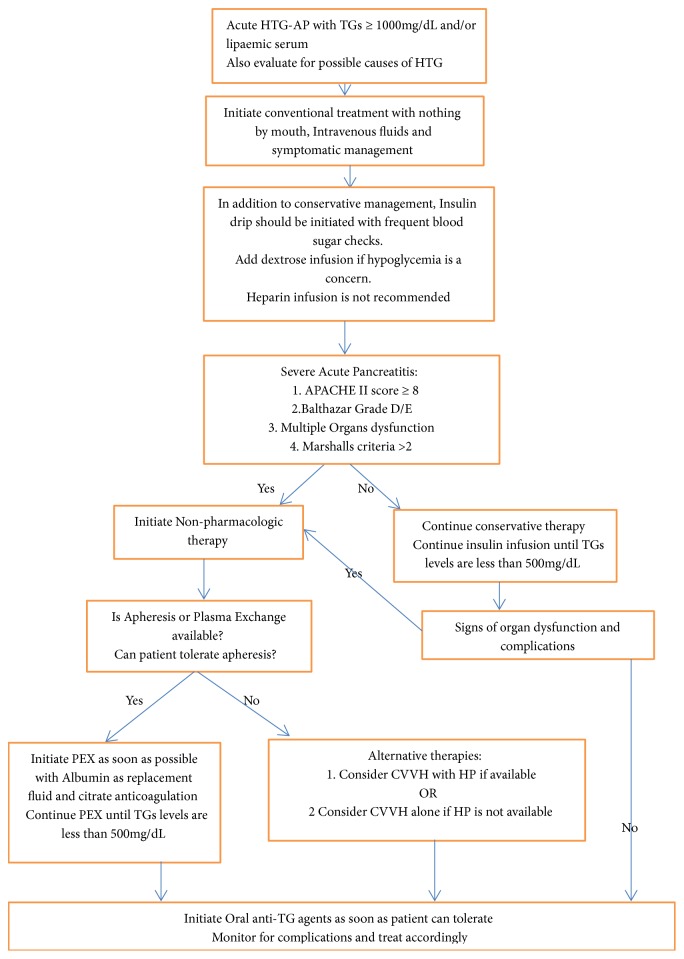
Proposed algorithm for treatment of HTG-AP.

HTG is more likely to be associated with severe pancreatitis as compared to other causes but no mortality difference has been reported. Recent data points to severe course of disease with higher TGs level on admission. Currently, there are no standardized treatment guidelines. We propose the following algorithm (Figure 1) based on review of current literature.

Initial therapy including bowel rest, intravenous fluids, and symptomatic treatment should be initiated. In addition, we also recommend insulin infusion at a rate of 0.1 to 0.3 U/kg per hour to lower TGs level irrespective of concurrent hyperglycemia. Frequent blood sugar checks every 1 hour should be done to prevent hypoglycemia and if present, dextrose solution should be added to infusion. If persistent hypoglycemia is encountered, insulin infusion should be stopped. TGs levels should be monitored every day to assess response to therapy.

Severe pancreatitis should be treated more aggressively. Patients with severe HTG-AP and those with APACHE II score ≥8, organ dysfunction, or Balthazar grade D/E should be offered intensive care unit and nonpharmacologic treatments. PEX with albumin as replacement solution is the most commonly used treatment modality. It should be initiated preferably within 36 hours provided patient is able to tolerate the treatment. There is some evidence that citrate anticoagulation might be better than heparin infusion for patient undergoing PEX. In centers where PEX is not available, consideration should be given to CVVH with HP in patients with SAP. CVVH alone should be considered in patients with SAP and organ dysfunction if PEX and HP are not available.

TGs lowering treatment should be continued until TGs < 500 mg/dL. Oral lipid lowering therapy should be started when patient is able to tolerate oral intake. Fibrates are the first-line recommended therapy. Aggressive lifestyle modifications should be done to prevent further episodes of acute pancreatitis. Patients with familial disorder should be counseled and TGs level should be monitored on a regular basis. While the above proposed treatment is consistent with reported literature, data on mortality benefit is lacking. Individual therapy should be tailored to each patient and their clinical condition. Further research efforts are needed to guide future therapies and uniform guidelines of this important clinical entity.

## Figures and Tables

**Table 1:** Studies evaluating role of plasma exchange in treatment of HTG-AP.

Author	Study design	Sample size	Age	M/F	Change of TG	Severity	Replacement fluid	Anticoagulation	Mortality	Comments
Lennertz et al. [58]	Retrospective case series	5	34	3/2	-74%	NA	Albumin	Heparin bolus 5/5, Heparin infusion 3/5, Citrate 2/5	0	Successful treatment of One pregnant patients

Chen et al. [44]	Retrospective comparison study	60 (only 20 patints received PEX)	42 ± 9	NA	-66%	Ranson score >3 in 10 patients	FFP (8) and albumin (12)	Heparin	4	No mortality benefit of PEX as compared to conservative treatment, no insulin described in control group

Yeh et al. [59]	Case series	18	27-65	13/5	-66%	NA	Albumin	Heparin	0	PEX better in lowering TG as compared to double filtration

Yeh et al. [60]	Retrospective study	17	31-53	10/7	-66%	Ranson score 2	FFP (8)/Albumin (9)	Heparin	2	Initiation of treatment at 3 days of symptoms onset, Anaphylactoid shock in one patient, 89% reduction in lipase levels

Kadikoylu et al. [61]	Case series	7	47±3	5/2	-46%	Asymptomatic	Albumin	Citrate	0	Asymtpomatic patients without any history of acute pancreatitis

Kyriakidis et al. [62]	Retrospective	10	42	8/2	-81%	Apache II 13	FFP	Citrate	1	

Al-Humoud et a [63]	Retrospective case series	8	34±9.19	6/2	-67%	NA	Albumin	Heparin	0	

Gubenšek et al [49]	Retrospective case series	50	45±8	46/4	-80%	APACHE II 5	Albumin	Heparin	6	42% mortality in patients with APACHE II score ≥ 8,

Stefanutii et al [46]	Retrospective multicenter case series	17	46 ± 10	9/8	-61%	NA/Resistant to conservative therapy	Albumin	Heparin bolus followed by Citrate infusion	0	12/17 patients had history of acute pancreatitis

Syed et al [64]	Retrospective case series	4	36	3/1	-89%	Apache II score 8	Albumin	NA	0	Fast lowering of TG but no clinical improvement

Ramírez-Bueno et al. [20]	Retrospective case series	11	40 ± 8	6/5	-81%	APACHE II Score 13 (9-18)	Albumin	NA	3	All 3 patients who died had APACHE II score > 15, Low TG levels in patients with severe pancreatitis

Gubenesek et al. [19]	Retrospective observational cohort study	111	47 ± 9	97/14	-59%	4 (2-7)	Albumin	Citrate (72) and Heparin (37)	6	5 cases of hypotension and 5 cases of hypocalcemia with citrate anticoagulation, no difference between early and late plasmapheresis, better outcomes with citrate vs heparin group

NA: not available, FFP: fresh frozen plasma, APACHE II score: Acute Physiology and Chronic Health Evaluation score.

**Table 2:** Studies summarizing the role of High Volume Hemofiltration (HVHF) in acute severe HTG-AP.

Studies/Parameter	Mao et al. [51]	Sun et al. [56]	He et al. [57]

Design	Interventional study	Controlled pilot study	Randomized controlled trail

Intervention	Blood purification (adsorption of triglyceride and hemofiltration), antihyperlipidemic agents (fluvastatin or lipanthyl), low molecular weight heparin (fragmin), insulin, topical application of Pixiao (a traditional Chinese medicine) over the whole abdomen within 72 hours of presentation	2 cycles of HVHF and HP of 24 hours each with 2 hours of HP within 48 hours in addition of standard treatment including insulin and heparin (Intervention) vs standard treatment (control)	Early HVHF with synthetic membrane without HP (Intervention) vs. Insulin and Heparin (control) group

Baseline parameter for severe acute pancreatitis	APACHE II score > 8 and serum TG s > 6.8 mmol/L	SAP based on Atlanta criteria with average APACHE of 14 in intervention group	SIRS 2/4, patients with multiple organ failure were excluded, average APACHE II score of 8

Sample Size	32	10	33

Primary end points	Resolution of symptoms	48 hours of intervention	Serum TGs level < 5.65 mmol/L

Outcomes	Significant reduction of APACHE II score, TNF-a and IL-10 after the therapy	Significant reduction of TGs levels, APACHE II score [5 vs 13.33 at 48 hours (p < 0.05)], inflammatory cytokines, SOFA score and reduced ICU stay [10 vs 16 days (p = 0.015)]	No significant reduction of APACHE II score or TGs reduction at Day 2 and 5

Benefits	No mortality in SAP	Reduced length of stay and severity of illness, improved outcomes	Faster lowering of TGs levels (9 hours vs 48 hours)

Complications	Less effective for fulminant SAP with APACHE II score >20 and signs of organ dysfunction	No reported complications	Significantly increased persistent organ failure rate (RR 2.42; CI, 1.15-5.11), double charges of hospitalization in intervention group

Limitations	Historic control group	Small sample size, no mortality difference reported	Baseline less sick patients, no HP used, Early stoppage of HVHF as TG levels were end points.

APACHE II score: Acute Physiology and Chronic Health Evaluation score; HVHF: High Volume Hemofiltration; HP: hemoperfusion; TGs: triglycerides; TNF-a: tumor necrosis factor a; IL: interleukin; SAP: severe acute pancreatitis; RR: relative risk; CI: confidence interval. SOFA: sequential organ failure assessment; SIRS: systemic inflammatory response syndrome.
